# A simple soil mass correction for a more accurate determination of soil carbon stock changes

**DOI:** 10.1038/s41598-023-29289-2

**Published:** 2023-02-08

**Authors:** Ames F. Fowler, Bruno Basso, Neville Millar, William F. Brinton

**Affiliations:** 1 Department of Earth and Environmental Sciences, Michigan State University, 288 Farm Ln, East Lansing, MI 48824 USA; 2 W.K. Kellogg Biological Station, Michigan State University, 3700 E Gull Lake Dr, Hickory Corners, MI 49060 USA; 3 Woods End Laboratories, 290 Belgrade Rd, Mt Vernon, ME 04352 USA; 4 School of Food and Agriculture, University of Maine, 168 College Ave, Orono, ME 04469 USA

**Keywords:** Agroecology, Carbon cycle, Carbon cycle, Climate-change mitigation

## Abstract

Agricultural soils can act as a sink for large quantities of soil organic carbon (SOC) but can also be sources of carbon to the atmosphere. The international standard for assessing SOC stock and measuring stock change stipulates fixed depth sampling to at least 30 cm. The tendency of bulk density (BD) to decrease with decreasing disturbance and increasing SOC concentration and the assumption of constant SOC and BD within this depth profile promotes error in the estimates of SOC stock. A hypothetical but realistic change in BD from 1.5 to 1.1 g cm^−3^ from successive fixed depth sampling to 30 cm underestimates SOC stock change by 17%. Significant effort has been made to evaluate and reduce this fixed depth error by using the equivalent soil mass (ESM) approach, but with limited adoption. We evaluate the error in SOC stock assessment and change generated from fixed depth measurements over time relative to the ESM approach and propose a correction that can be readily adopted under current sampling and analytical methods. Our approach provides a more accurate estimate of SOC stock accumulation or loss that will help incentivize management practice changes that reduce the environmental impacts of agriculture and further legitimize the accounting practices used by the emerging carbon market and organizations that have pledged to reduce their supply chain greenhouse gas (GHG) footprints.

## Introduction

Agriculture provides food, feed, fuel, and fiber to sustain our lives on the planet. While providing critical ecosystems services, agriculture also contributes to climate change, being responsible for 23% of the global greenhouse gas (GHG) emissions^1^. Because agriculture is the dominant sector affecting landscape management and soil resources, good agricultural policy is needed to mitigate the impact of climate change by reducing GHG emissions, sequestering carbon in the soil, and building resilience against now unavoidable climate impacts^2^. Private industry and non-profit organizations are rapidly developing voluntary carbon markets to value increases in carbon in agricultural soils. Nations are likewise committed to measuring soil organic carbon (SOC) stocks; 28 countries have explicitly committed to increasing SOC within their Nationally Determined Contributions in the Paris Agreement^3^. Protocols for the measurement, reporting and verification (MRV) of SOC are largely ununified and produce nontransferable credits^4^. For these reasons, there is a pressing need to properly quantify SOC stocks for reporting and verification requirements and to ensure that high-quality credits are issued to the market and traded for effective and realistic net atmospheric C sequestered^5^.

Several protocols have been developed by various C market organizations to quantify SOC credits generated on cropland and rangeland based on different approaches to determine SOC and GHG removals. These rely on soil sampling, modeling, or a hybrid approach with modeling, sampling, and remote sensing^4,6^. Protocols are trending towards a hybrid MRV approach based on SOC modeling and using data from samples taken prior to a management change and periodic re-sampling afterwards (e.g., 5 years) to re-calibrate model estimates and scale MRV^6,7^. The desire to reduce the number of soil samples and their processing and analysis stems from the current small margins between the revenue generated from the sales of carbon credits and the cost of sampling and analysis to adequately confirm changes in SOC stocks^8^. Paying farmers to sequester C remains a tentative approach to climate change mitigation due to the uncertainties of accurately observing, much less modeling changes in SOC stocks over time and confirming their permanence^9^.

Soils, and SOC stocks in particular, are prone to high spatial heterogeneity. Commonly, the coefficient of variance of a test trait determines the nominal sample size to attain a specific level of precision^10^. Compositing samples may reduce standard error, sampling cost, and, if well designed, improve geospatial models of SOC^8,11^. Defining the spatial unit defined by a soil sample remains a challenge, and there is considerable error in measuring SOC in each sample and at larger scales^12^. The conversion of the well-established SOC concentration term (mass of SOC stock (kg)/total mass of the soil (kg) to an SOC stock (kg C ha^−1^) is commonly obtained by multiplying the carbon percentage with the bulk density (BD—expressed as the mass of a relatively undisturbed soil and pore space within a known volume of sample), of a 30 cm fixed-depth sample, and a unit conversion constant^4,13^. The 30 cm fixed depth sample is a minimum standard set by the IPCC, though deeper sampling (e.g., 100 cm) is recommended where possible^6^. The measurement of BD is also prone to sampling error, often greater than that of SOC and must be corrected for stone content^14^. A typical overall random error may be 7–8%^15^, but larger errors (10–40%) in BD estimation are not uncommon^16^.

**Figure 1:**
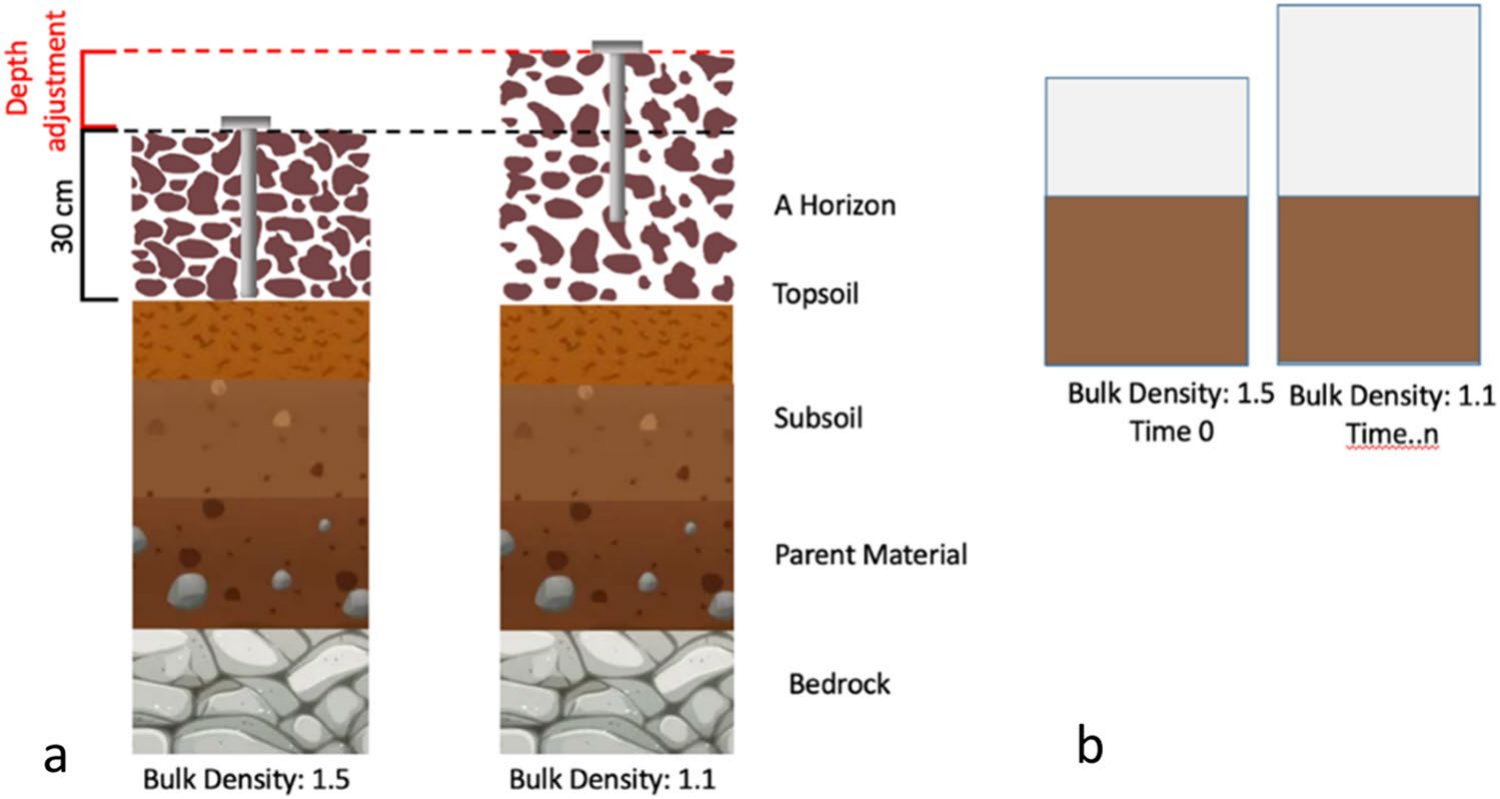
(**a**) Example of pedon alteration due to changes in bulk density over time showing the position of the soil sampling device from the soil surface to the same fixed depth. (**b**) Example of pedon after the correction showing the difference in air space, while the soil mass remains constant.

Determining a rate of change in SOC stock (kg C ha^−1^ year^−1^) requires repeated sampling at the same site over time. While this static, synchronous approach presents a risk of preferential management of fixed sample locations^17^, increased sampling efficiency by limiting spatial variance, minimizes the cost of field work, is commonly accepted as standard^18^ and predominates over other synchronous or rotational sampling schemes on a field-by-field basis^19^. Bulk densities change with land use change^20–22^ and conservation agriculture (i.e., reduced tillage, surface mulching, cover cropping and diverse rotations)^23^. Tillage and large inputs of organic matter over time have been shown to substantially decrease BD in grain crop systems. Celik et al. found a 20% decrease in BD over 12 years following application of compost when compared to mineral fertilizer^24^, and Gál et al. measured a 17% decrease in BD over 27 years when moldboard plow was used to till when compared to no-till^25^. Taking successive fixed depth samples where increased soil carbon and soil aggregation has reduced BD will result in a smaller soil mass observed in the second sample. The BD is calculated as the relationship between the bulk, fine dry mass, and volume of a sample $$BD =\frac{{M}_{T}}{V}$$; often shown in mass per area terms $$BD =\frac{{M}_{TA}}{D}$$ (i.e., Mg ha^−1^), where *D* is soil depth, SOC concentrations are measured by total soil mass per area ($${M}_{TA}$$) and the SOC mass concentration, $$SO{C}_{stock}= {M}_{TA}*SOC$$ or more commonly $$SO{C}_{stock}= BD*D*SOC$$. The direct calculation of an SOC stock with a changing BD will introduce sample error when sampled to a fixed depth because different soil masses are sampled. A reduction in BD due to better management (e.g., greater porosity because of improved aggregate stability and decompaction) or the improper timing of sampling following for example a tillage event, could lead to a calculated loss in SOC stock. This would be observed if the increase in SOC was exceeded by the ‘loss’ of soil and associated SOC following expansion below the fixed sample depth^20,26^. The inverse effect may be expected with an increase in the BD of soil. Fixed-depth measurements of SOC could therefore withhold credits to farmers where they are warranted, i.e., in a field that now has a lower BD and higher SOC content (false negative) and award credits where they are not warranted for an adjacent field that now has a higher BD and lower SOC content (false positive) (Fig. 1).

Correcting for fixed depth measurements of soil components impacted by changes in soil BD by using fixed soil mass per unit area is called the equivalent soil mass (ESM) approach. Taken cumulatively on SOC stock on a fine soil mass basis allows for interpolation to a standard mass and is called the material coordinate approach. Using the first fixed depth sample mass as the baseline in a static synchronous sampling scheme is referred to as the initial or original ESM approach^27,28^. Soil mineral mass rather than soil total mass is used as it is much less sensitive to changes in soil volume, as changes in the less dense soil organic matter (SOM) over time will change the total mass in a sample^29^. Soil mineral mass can be measured directly or calculated as:

1 $${M}_{M}={M}_{T}*\left(1-\mathrm{k}*\mathrm{SOC}\right)$$

where M_T_ is the soil total mass, M_M_ is the soil mineral mass, SOC is the concentration (%), and k is the van Bemmelen factor that relates the total mass of organic C in the soil to the SOM. Here we use a k value of 1.9 by assuming that SOM contains 53% carbon^30,31^.

The ESM approach for correction of SOC has been reviewed extensively in the literature, much of which has supported its adoption in place of the standard fixed depth approach^25,29,32,33^. Notable recent work on ESM theory by von Haden et al., and an empirical review of the ESM effect on changing land use by Rovira et al. further promotes the case for the broad adoption of ESM^20,34^. Despite these findings, the ESM approach has seen very limited uptake by soil sampling and analytical laboratories and therefore by extension in organizations and in processes, such as MRV platforms in carbon markets, that use and rely on SOC stock measurements for policy and accounting decisions^4^.

Data on total sample mass or mineral soil mass, needed for ESM, is rarely recorded and is typically only available as a reconstruction from depth and BD values. To this end, von Haden et al. created an R package to convert values from smaller (< 30 cm) sample core depths^34^. In this paper, we evaluate the error in SOC stock assessment generated from fixed depth sampling relative to an equivalent soil mineral mass (ESM) approach. We propose straightforward post hoc mass correction terms based on linear interpolation to the original sample mass that can be routinely adopted under current, 30 cm fixed depth sampling schemes and analytical data output procedures. We also determine the error introduced from the assumption of a constant SOC concentration and BD value with depth in a fixed depth sample and suggest alternative sampling strategies that reduce this error.

The main objectives of this research are to: (1) demonstrate the advantage of using the ESM approach as opposed to the fixed depth approach when measuring SOC stock changes over time, (2) present simple ESM corrections that are compatible with current soil sampling protocols and can be integrated directly into current laboratory data work flows, and (3) demonstrate the improved accuracy of SOC stock change estimation when shorter sample depth intervals are taken and where SOC and BD vary realistically with depth. Our approach provides for more accurate reporting of carbon accumulation or loss in soil that is urgently needed for improved SOC stock accounting in carbon markets, research, and other organizations, and in policy and finance discussions that can promote a more sustainable and socially responsible investment to reduce the impacts of climate change.

## Methods

Our approach uses hypothetical 30 cm fixed depth samples taken at three successive time points (t0, t1, and t2) with prescribed changes in SOC (1.4% to 1.6%) and BD (1.5–1.1 g cm^−3^) over these time points (Table 1). The 30 cm soil depth is the common international standard for sampling and analysis required for SOC stock assessment and adhered to by carbon accounting and market organizations^6,18^. The changes we adopted (a 27% decrease in BD and a 14% increase in SOC) while relatively large, are consistent with those reported in the literature. For example, Reganold and Palmer reported a 25% decrease in BD (1.2–0.9 g cm^−3^) in neighboring farms with differing management practices^23^, and Syswerda et al. observed a 17% increase in SOC concentration (10.4–12.2 g C kg soil^−1^) when converting from a conventionally to organically managed row crop rotation^21^.

**Table 1:** Hypothetical changes in bulk density (BD) and soil organic carbon (SOC) concentration in 30 cm fixed depth samples at time points t0, t1 and t2 along with calculated values of SOC stock and total soil mass and mineral soil mass.

Time	30 cm fixed depth samples		
	t0	t1	t2
Bulk density (g cm^−3^)	1.50	1.30	1.10
SOC (%)	1.40	1.50	1.60
Depth of layer (cm)	30	30	30
Total soil mass (Mg ha^−1^)	4500	3900	3300
Mineral soil mass (Mg ha^−1^)	4380	3789	3200
SOC stock (Mg ha^−1^)	63	59	53

In Table 1, the total soil mass, mineral soil mass, and the SOC stock of the fixed depth samples were calculated by equations as described in the introduction from our prescribed changes in BD and SOC values.

### Scenarios

**Figure 2:**
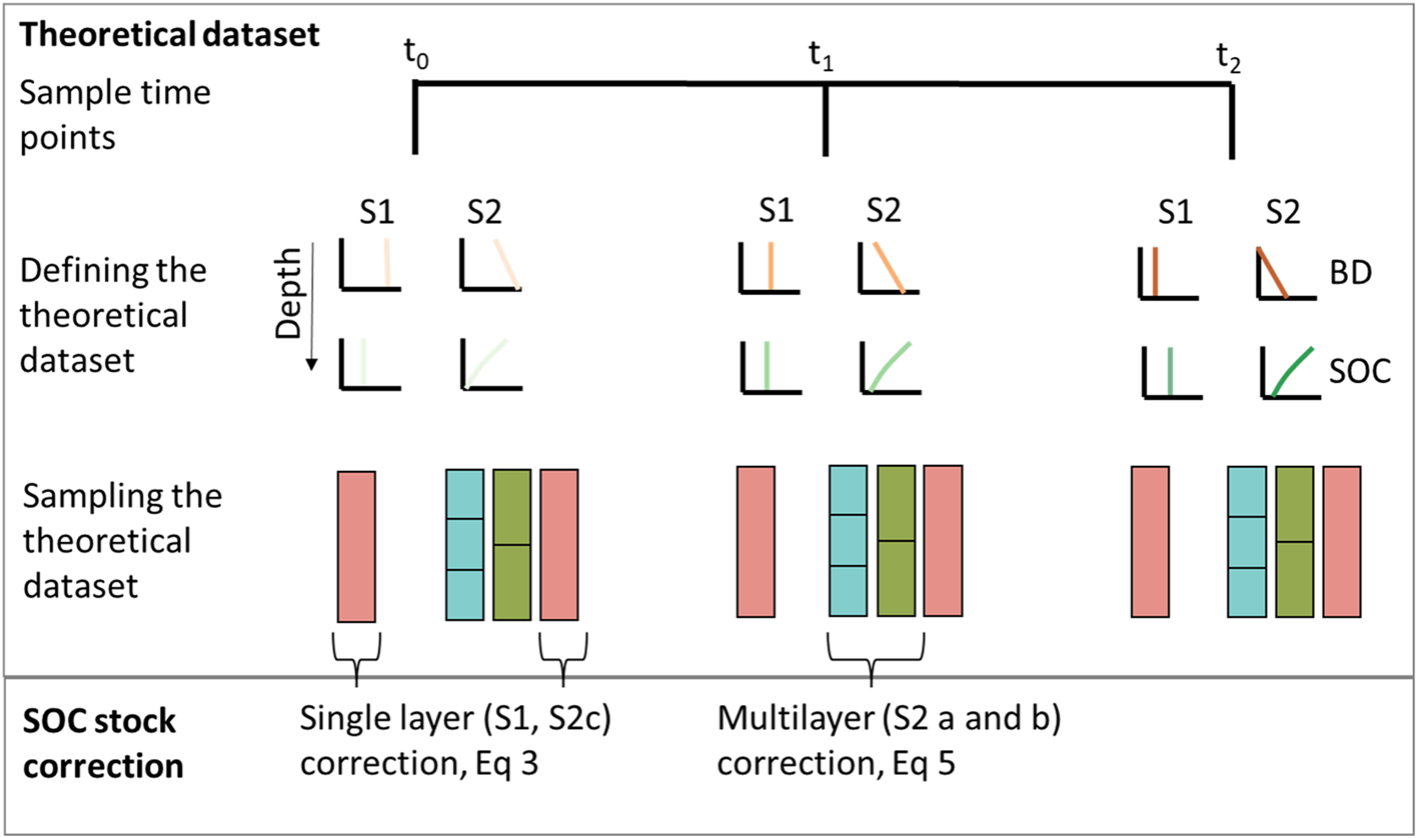
Flow chart of the definition, sampling, and SOC stock correction for a theoretical data set at time points t0, t1, and t2 for scenarios s1 with linear distributions of BD and SOC and s2 with a linear increase in BD and exponential decrease in SOC with depth. Scenario s2 is sampled at (**a**) 10 cm, (**b**) 15 cm, and (**c**) 30 cm intervals.

**Figure 3:**
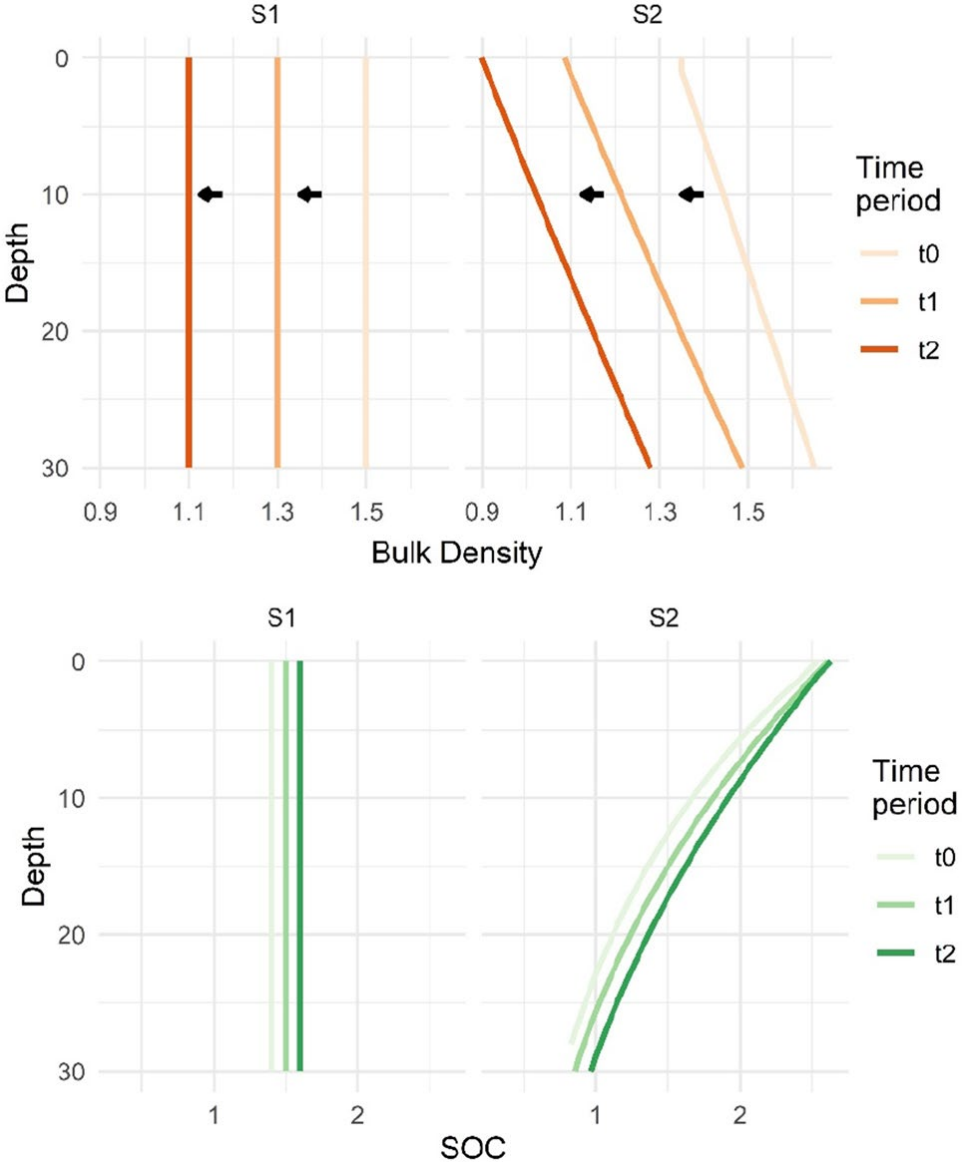
Scenarios (S1 and S2), showing (**a**) bulk density variation (BD, g cm^−3^), and (**b**) soil organic carbon (SOC, %) variation by depth (0–30 cm) at each time point (t0, t1, and t2). For scenario 2, the single 30 cm depth interval was used (2c). See Table 1 and 2 for details.

We compared hypothetical ESM correction scenarios with our 30 cm fixed depth sample at each time point (Table 2, Figs. 2, 3).

**Table 2:** Hypothetical ESM scenarios showing variation with depth for bulk density (BD) and soil organic carbon (SOC) at each sampling time point, along with the sample depth intervals investigated.

Scenario	BD by depth	SOC by depth	Sample intervals (cm) and [number]
1	Constant	Constant	30 [1]
2a	Linear decay	Exponential decay	10 [3]
2b	Linear decay	Exponential decay	15 [2]
2c	Linear decay	Exponential decay	30 [1]

#### Scenario 1

We carried out the ESM correction on a 30 cm sample and assumed that the sample was homogenous throughout the profile, with constant SOC and BD values at each time point.

To correct for the error in SOC stock estimation when using fixed depth soil sampling, we used  Eqs.2a, 2b and 2c that consider changes in BD^28,35^. The adjusted soil depth resulting from the change in BD is calculated as:

2a $${\mathrm{M}}_{\mathrm{n}}= {\mathrm{M}}_{\mathrm{i}}$$

2b $${\mathrm{D}}_{\mathrm{a}}*{\mathrm{BD}}_{\mathrm{n}}*\left(1-\mathrm{k}*{\mathrm{SOC}}_{\mathrm{n}}\right)={\mathrm{D}}_{\mathrm{i}}*{\mathrm{BD}}_{\mathrm{i}}*\left(1-\mathrm{k}*{\mathrm{SOC}}_{\mathrm{i}}\right)$$

2c $${\mathrm{D}}_{\mathrm{a}}={\mathrm{D}}_{\mathrm{i}}*\frac{{\mathrm{BD}}_{\mathrm{i}}}{{\mathrm{BD}}_{\mathrm{n}}}*\frac{1-\mathrm{k}*{\mathrm{SOC}}_{\mathrm{i}}}{1-\mathrm{k}*{\mathrm{SOC}}_{\mathrm{n}}}$$

where M_i_ = Initial mineral soil mass per area $\left[\frac{M}{{L}^{2}}\right]$ , M_n_ = New mineral soil mass per area $\left[\frac{M}{{L}^{2}}\right]$ , D_a_ = Adjusted soil surface depth $\left[L\right]$ , BD_i_ = Initial bulk density $\left[\frac{M}{{L}^{3}}\right]$ , BD_n_ = New bulk density $\left[\frac{M}{{L}^{3}}\right]$ , SOC_i_ = Initial SOC as a decimal percent $\left[\frac{M}{M}\right]$ , SOC_n_ = New SOC as a decimal percent $\left[\frac{M}{M}\right]$ , D_i_ = Initial depth $\left[L\right]$.

To conform with Eq. (2a), an increase in SOC over time results in a displacement of some soil mineral mass from the sample, whereas a decrease in SOC over time requires some soil mineral mass to be replaced^34^. Multiplying the BD by the mineral fraction of the soil $\left(1-\mathrm{k}*{\mathrm{SOC}}\right)$ for each time point allowed us to compare equivalent mineral mass^28^. The effect of a change in SOC on mineral mass is small, with a 1% change in SOC equating to approximately a 2% change in apparent depth. This adjustment relates SOC per unit of mineral mass of the fine fraction (< 2 mm) and is unaffected by the coarse fraction (> 2 mm)^20^. The corrected apparent depth can then be used to calculate the corrected SOC stock of a single layer, fixed depth sample (Eq. 3).

3 $$SO{C}_{stock}={D}_{a}*BD*SOC$$

#### Scenario 2

In ESM correction scenarios 2a, 2b, and 2c, we imposed variable, dynamic BD and SOC values with depth over time (Table 2, Figs. 2, 3). To investigate these profiles, we determined the SOC and BD values throughout the soil depth by separating the soil into one (1) cm depth increments (i.e., 0–1 cm, 1–2 cm, etc.). We refer to this calculated incremental profile as the scenario 2 baseline. We assumed that our prescribed SOC concentration varied with depth following an exponential decay. To represent this decay, we simulated the global average distribution of SOC concentration with depth on crop land^36^, following the distribution from Hobley and Wilson^37^ (Eq. 4),

4 $$SOC\left(d\right)=SO{C}_{\infty }+\left(SO{C}_{o}-SO{C}_{\infty }\right)\times {e}^{-dk}$$

where *SOC *(*d*) is the SOC concentration at depth (d), ${SOC}_{\infty }$ is the infinity SOC concentration, *SOC*_*0*_ is the SOC concentration at the soil surface, and k is the decay rate. We solved for the decay rate, initial *SOC*_*0*_, and infinity ${SOC}_{\infty }$ to fit the global average distribution for the 30 cm profile^36^ and then scaled the SOC concentration to our 30 cm fixed depth sample’s average SOC (1.4%) at t0 (Fig. 3).

In scenarios 2a, 2b, and 2c, the BD increased linearly with depth^38,39^. At the initial time point (t0), we varied the BD values by ± 10% of the BD average over the 30 cm depth, such that for example, BD at t0 (profile average of 1.5 g cm^−3^) was 1.35 g cm^−3^ and 1.65 g cm^−3^ for the upper (0–1 cm) and lower (29–30 cm) depth increment, respectively. For each sequential time point, as the average BD decreased, the soil expanded. To determine the expansion, the depth of the initial sample (e.g., at t0) that filled the 30 cm depth in the subsequent sample (e.g., at t1) was calculated as the initial depth multiplied by the ratio of the average initial BD over the average new BD (e.g., 1.5/1.3 = 1.15 for t0/t1).

The linear increase in BD with depth of each following time point maintained the average BD of scenario 1. We then varied the new BD by ± the percent change in the average BD between the time periods (see annotated scripts “main.R” and “functions.R” in Supplementary Material 1 for the development of the theoretical dataset). We then divided each initial BD increment (using soil mass for every 1 cm depth increment) by the new BD in the expanded increment (using soil mass for every > 1 cm depth increment) to determine the expanded depth of each increment. The SOC value at the initial time represented the same, now expanded, (> 1 cm) increments, as SOC is a ratio of mass. We used a linear decay rate that was twice that of the percent change in BD between time points to maintain an average BD that was consistent with scenario 1. To model the subsequent fixed depth sample, the BD and SOC concentration values of this expanded soil profile were then interpolated back to the 30 × 1 cm increments of the scenario 2 baseline depth. This calculation preserved the prescribed average BD of the new time point by only expanding the initial SOC concentration.

We adjusted the SOC concentration of the next time point to maintain the average SOC concentrations of the 30 cm fixed depth sample, (see annotated scripts “main.R” and “functions.R” in Supplementary Material 1). Because the BD changed between time points and because the SOC stock in the 30 cm fixed depth sample was known, we determined the change in SOC stock between time points by subtracting the average SOC stock in the prior sample from the new sample. We then weighted this change across the 30 cm profile using the distribution of the global soil SOC in the top 30 cm to simulate SOC stratification with reduced tillage or agricultural intensification^40^. We then multiplied this change by the BD to convert back to SOC concentration and added the delta $(\Delta )$ SOC value to the prior sample. A worked example is shown in Supplementary Material 2 “Correction Example”.

At each time point we split the soil profile at 10 cm and 15 cm depth intervals to create samples for scenarios 2a (3 soil intervals) and 2b (2 soil intervals), respectively. Note that scenario 2c is mathematically equivalent to scenario 1—with only one sample depth interval (30 cm) the sample contains no data on varying SOC or BD. The samples for 2a and 2b were generated by summing the total mass per area and SOC stock values from the scenario 2 baseline to produce single sample values of total soil mass per area and SOC concentration values per depth interval (as would be determined in a laboratory) and calculating BD and mineral mass.

In scenario 2, any required additional mineral mass and the associated SOC values were ‘placed’ at the base of the sample to represent a soil profile that had expanded below the fixed 30 cm depth. To account for this, we calculated the increase in adjusted sample depth and accumulated additional soil mineral mass with the lowest sample depth interval of each split sample (Eqs. 5 and 6).

5 $$\mathrm{\Delta D}={D}_{a}-{D}_{i}$$

6 $$SO{C}_{stock}={(D}_{1}*B{D}_{1}*SO{C}_{1}+\dots + {(D}_{j}+\Delta D)*B{D}_{j}*SO{C}_{j}))*10^2 (\mathrm{g}/\mathrm{cm}^{2})/(\mathrm{Mg}/\mathrm{ha})$$

where $\mathrm{ \Delta D}$ is the apparent change in depth needed to generate the same mineral mass of the initial sample and the subscript j is the number of sample depth intervals from 1 to j.

Varying BD linearly with depth introduces additional complexity in the calculation of the apparent depth. Each sample depth interval may expand (or contract in cases not explored here) at differing rates. Here, the over or under sampling of soil mineral mass is no longer constant with depth and the correction for apparent depth (Da) is estimated with linear interpolation using the BD of each sampling depth interval (i.e., 10 cm, 15 cm, or 30 cm). To do so, we calculated the mineral mass in each depth interval, determined their difference between the initial sample time point and new sample time point, and converted the change in mineral mass to a depth, where:

7 $${\mathrm{D}}_{\mathrm{a}}={\mathrm{D}}_{\mathrm{i}}+\frac{\left(\mathrm{sum}\left({\mathrm{D}}_{\mathrm{ij}}*{\mathrm{BD}}_{\mathrm{ij}}*(1-\mathrm{k}*{\mathrm{soc}}_{\mathrm{ij}}\right))- \mathrm{sum}\left({\mathrm{D}}_{\mathrm{nj}}*{\mathrm{BD}}_{\mathrm{nj}}*(1-\mathrm{k}*{\mathrm{soc}}_{n\mathrm{j}})\right)\right)}{{\mathrm{BD}}_{{\mathrm{nj}}_{\mathrm{bottom}}}*1-\mathrm{k}*{\mathrm{soc}}_{n{\mathrm{j}}_{\mathrm{bottom}}}}$$

where j_bottom_ is the lowest sample depth interval, and other terms are as previous. Using Eqs. (5), (6), and (7), with variable BD and SOC values, SOC stock can be corrected using samples split into the 10 cm and 15 cm sampling depth intervals.

## Results

### Scenario 1

When using the 30 cm fixed depth sample for the non-ESM corrected sample, our hypothetical increase in SOC concentration (from 1.4 to 1.6%) resulted in an apparent loss of SOC stock from 63.0 to 52.8 Mg ha^−1^ (16.2%; Table 3). For the ESM correction, soil depth was corrected for the influence of BD on fixed depth sampling to give the adjusted soil depth (Da) that extends below the original 30 cm depth (34.7 cm and 41.1 cm at t1 and t2, respectively) and results in a corrected gain in SOC stock from 63.0 to 72.3 Mg ha^−1^ (14.8%), an increase of 37% above the estimated SOC stock from the fixed depth approach at t2.

**Table 3:** Scenario 1: Changes in SOC stocks, total soil mass, and mineral soil mass mass using the fixed depth and adjusted depth (ESM correction) approach at three time points (t0, t1, and t2).

Time point	30 cm fixed depth sample			Adjusted 30 cm depth		
	t0	t1	t2	t0	t1	t2
Bulk density (g cm^−3^)	1.50	1.30	1.10	1.50	1.30	1.10
Corrected sample depth (cm)	30	30	30	30	34.68	41.07
Total soil mass (Mg ha^−1^)	4500	3900	3300	4500	4508	4517
Mineral soil mass (Mg ha^−1^)	4380	3789	3200	4380	4380	4380
SOC (%)	1.40	1.50	1.60	1.40	1.50	1.60
SOC stock (Mg ha^−1^)	63.0	58.5	52.8	63.0	67.6	72.3
SOC stock change (%)	–	− 7.1	− 16.2	–	7.3	14.8

### Scenario 2

Between time points t0 and t2, the corrected SOC stock increased in scenarios 2a and 2b (10 cm and 15 cm sample depth intervals, respectively) by 3.7% and 7.9%, respectively (Table 4). Again, note that scenario 2c is equivalent to scenario 1 (both 14.8% SOC stock increase)—with only one sample depth interval (30 cm) the sample contains no data on varying SOC or BD, and so data is not shown. Therefore, separating the soil sample (30 cm) into depth intervals (10 cm or 15 cm increments) reduced the increase in SOC stock when compared to the full 30 cm sample, approximately halving and quartering the increase in the 15 cm and 10 cm intervals, respectively.

**Table 4:** Scenario 2: Changes in SOC stocks, total soil mass, and mineral soil mass using adjusted depth (ESM correction) approach, with split sampling intervals of 10 cm (scenario 2a) and 15 cm (scenario 2b) intervals at three-time points.

Time point	Adjusted depth 2a: 10 cm samples			Adjusted depth 2b: 15 cm samples		
	t0	t1	t2	t0	t1	t2
Bulk density (g cm^−3^)	1.5	1.3	1.1	1.5	1.3	1.1
Average SOC (%)	1.4	1.5 (1.04)*	1.6 (1.16)*	1.4	1.5 (1.13)*	1.6 (1.27)*
Corrected sample depth (cm)	30	34.49	40.09	30	34.61	40.38
Total soil mass (Mg ha^−1^)	4500	4503	4507	4500	4504	4509
Mineral soil mass (Mg ha^−1^)	4380	4380	4380	4380	4380	4380
SOC stock (Mg ha^−1^)	63.0	64.6	66.7	63.0	65.2	68.0
SOC stock change (%)	–	2.5	3.7	–	3.5	7.9

*Indicates the average SOC concentration in the lowest sample depth interval.

**Figure 4:**
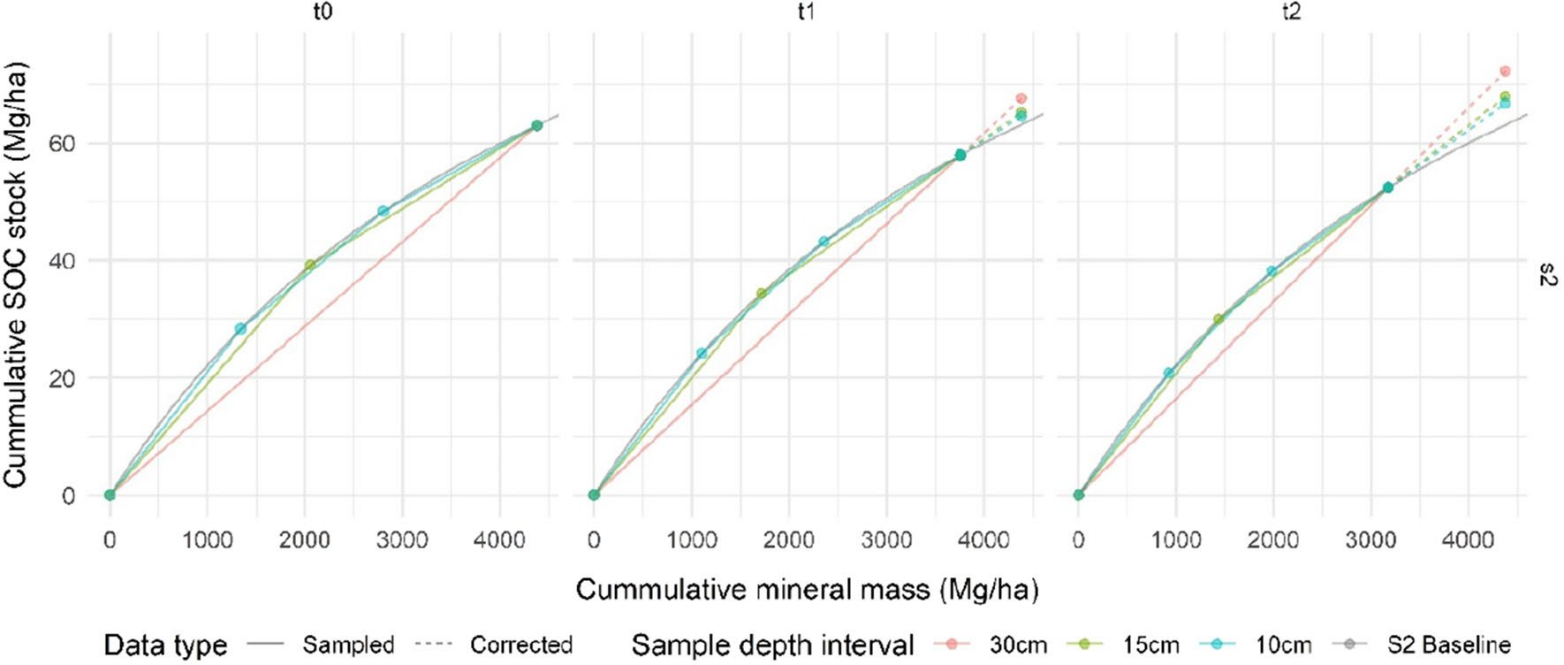
Corrected cumulative SOC stock (Mg ha^−1^) per cumulative mineral mass for scenario 2 at each time point for sample depth intervals 10 cm, 15 cm, and 30 cm (scenarios 2a, 2b, and 2c, respectively) compared to the scenario 2 baseline profile (grey). Sample lines (solid) converge on the 30 cm interval sample depth with the 30 cm sample line (orange) equivalent to scenario 1. Data type is shown by line type where sampled data are solid and corrected data are dashed.

**Figure 5:**
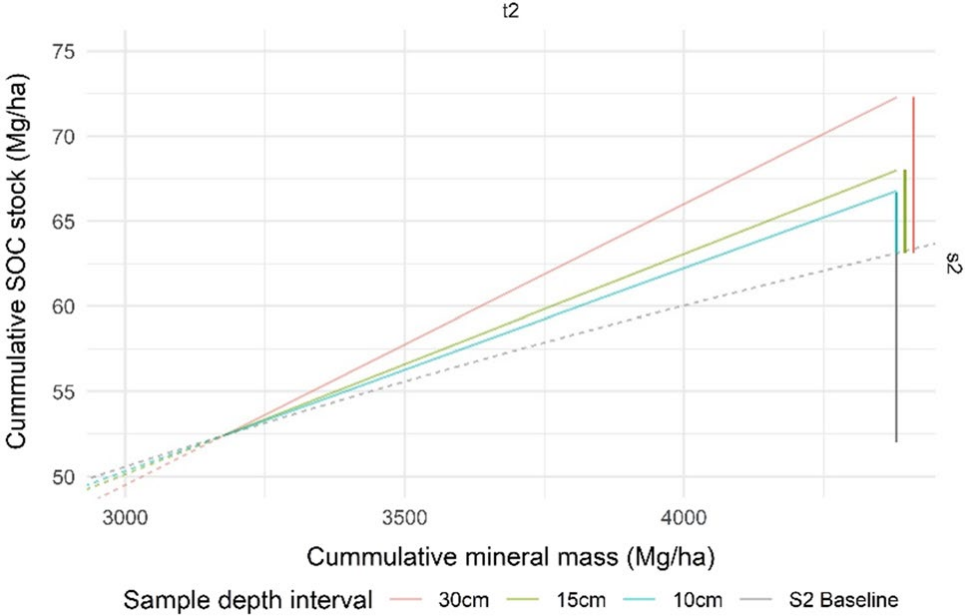
Corrected cumulative SOC stock (> 50 Mg ha^−1^) per cumulative mineral mass (> 3000 Mg ha^−1^) for scenario 2 at time point t2 for sample depth intervals 10 cm, 15 cm, and 30 cm (scenarios 2a, 2b, and 2c, respectively) compared to the scenario 2 baseline profile, showing vertical error bars due to no correction with changing bulk density. The error is the difference in the corrected SOC stock from the global average soil at the same mineral mass. Sampled data are solid and corrected data are dashed as for Fig. 4.

**Figure 6:**
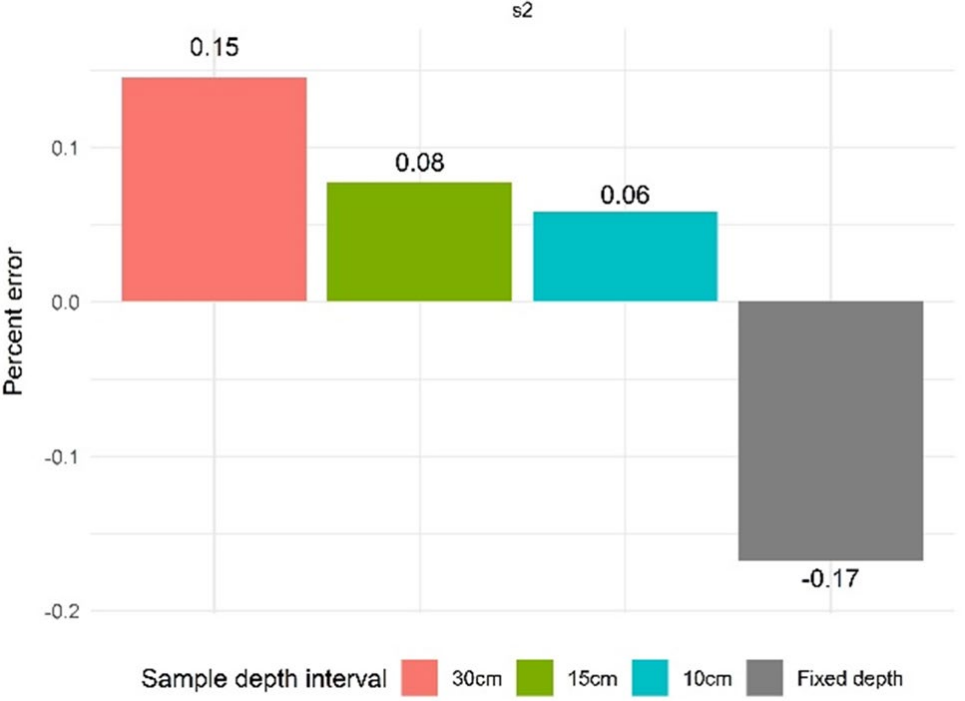
Correction errors (%) at time point t2 for sample depth intervals 10 cm, 15 cm, and 30 cm (scenarios 2a, 2b, and 2c, respectively) compared to the scenario 2 fixed depth baseline.

The error in the linear interpolation correction is defined by the percent difference between the correction and the scenario 2 baseline (incremental profile; see “Methods”). Figure 4 shows the modeled cumulative SOC stock variation with the cumulative mineral mass for scenario 2 at the three sample depth intervals, alongside the scenario 2 baseline profile. The sample points represent the values of each sample depth interval (i.e., 10 cm, 20 cm, and 30 cm for scenario 2a, 15 cm and 30 cm for scenario 2b, and 30 cm for scenario 2c), whereas Tables 3 and 4 present the cumulative sampled or corrected values for each time point. The scenario 1 value is identical to scenario 2c at all-time points, so is not shown here. Using time point t2 as an example, the correction errors for SOC stock and for combined % error for scenario 2 are shown in Figs. 5 and 6, respectively.

Figure 6 compares the direction and magnitude of the combined error between the 30 cm fixed depth sample and the three depth intervals for scenario 2 at time point t2. Scenario 2 correction errors decrease with decreasing sample interval depth (15%, 8%, and 6% for the 30 cm, 15 cm, and 10 cm intervals, respectively). The % error for the 30 cm sample interval is of similar magnitude but different direction, to the % error for the fixed depth sample (17%) for SOC stock change estimation.

## Discussion and recommendations

The agricultural industry is poised to aggressively increase SOC stocks through regenerative practices such as no-tillage, cover crops, and altered fertilizer inputs as incentivized by carbon and environmental markets, but error in MRV and changing BD in particular requires simple and standard correction^16^. The standard soil sampling for SOC stocks contain all the needed data for the ESM correction, though depth stratification is needed to reduce overfitting. We have provided an example Excel spreadsheet with single and multiple depth interval samples to demonstrate the ease of systematic integration of this correction into soil laboratory processes (see Supplemental Material 2).

For the 30 cm single depth profiles, our ESM correction overestimated SOC stocks to a similar degree that the fixed depth approach underestimated the SOC stocks (Fig. 6). The contrast between our SOC stock change estimates (30 cm single depth) when using the fixed depth (− 16%) or ESM (+ 15%) approach highlights their inherent divergence and the profound implications of the choice of method used, particularly when applied at scale. As a theoretical example, if we assume that the 61 million hectares of corn and soybeans typically planted annually in the U.S. Midwest^41^ were to experience (due to various changes in management practice) a reduction in soil compaction as prescribed here (i.e., a decrease in BD from 1.5 to 1.1 g cm^−3^), then estimates of SOC stock change would indicate a loss of 0.62 gigatons (fixed depth) or a gain of 0.57 gigatons (ESM). The divergent messages that these results confer would no doubt strongly influence farmer incentive (or lack thereof) to change practice. Given that the focus of many carbon market initiatives in agriculture relate primarily to SOC sequestration for payment^5^, accurate MRV will dramatically alter the role that agriculture may play in mitigating climate change.

Correction error can be drastically reduced with depth stratification. When 30 cm ESM samples were split, the smaller depth intervals resulted in a decrease in SOC stock overestimation and a large reduction in correction error percentage, with the 15 cm depth intervals halving the error. An increase in SOC stock was found for all the ESM correction scenarios used here; a directionality that can also be viewed as positive with regards to messaging for practice change, and that was agnostic to changes in SOC concentration and BD variability within the full depth profile and the depth intervals into which the 30 cm sample profiles were split. While the study does not cover all potential variation of the interdependency between soil depth, soil mass, BD, and SOC, our multi-scenario approach and results encompass this underlying complexity and allow us to evaluate a straightforward EMS correction. The accurate accounting of SOC stock change using the ESM approach and clear recommendations on its uses and limitations is vital to help promote good management, but also to avoid an incentivization of practice changes that do not increase SOC, and could lead to unwarranted financial rewards and a sense of accomplishment. The recommendations and simple ESM correction developed here for SOC stock calculations can be incorporated into routine sampling and analytical protocols. This is described in more detail below.

Post hoc ESM corrective approaches to fixed depth sampling have previously been developed^20,34^. While robust, they require sampling at multiple depths or genetic horizons and the use of more complex mathematical relationships between soil parameters. Both of which are time, cost, and expertise constrained, and impediments to broader use. Our corrections work with typical 30 cm fixed depth sampling, readily undertaken and analyzed by field and laboratory technicians to provide standardized, comparable data most frequently requested and understood by multiple stakeholders.

Splitting sample cores into multiple, smaller depth increments can produce a more accurate and precise estimate of SOC stock. It may however be cost prohibitive for standardized sampling at large geographic scales^8^. Our work shows that a sample split into two sample depth intervals (0–15 cm and 15–30 cm) at representative locations, while requiring some additional soil processing and analysis can be a workable trade-off, given the much-improved accuracy of SOC stock estimate when using our ESM correction as compared to a single fixed depth. Evaluating the per area cost of soil sampling at scale varies by the number of samples needed to achieve sufficient statistical power. The cost is also dependent on many other human and organizational variables such as sampling region, available personnel and their expertise, suitable equipment, and other resources, as well as travel time to sites and associated costs. Minimizing the labor and cost required to generate accurate, reproducible results is essential. Our ESM correction is presented to align with current fixed depth sampling procedures and can be adopted for direct comparison of SOC stock per mineral mass of the soil (see Supplementary Material 2). Our recommendations when sampling at fixed depth are therefore:

1. Record the sample mass and accurate depth, along with sampling tool internal diameter (for sample volume determination) to avoid the use of measuring BD in future samples.
2. When only homogenous, 30 cm fixed sample (no splitting and without mass) data are available, incorporate Eqs. (2a–2c) and (3) into the laboratory data output process (Supplementary Material 2). Understand that SOC stock may be overestimated.
3. When split samples (e.g., 0–15 cm and 15–30 cm) from 0 to 30 cm fixed samples are available, incorporate Eqs. (5–7) into the laboratory data output process (Supplementary Material 2)
4. To reduce ESM correction error, e.g., for research purposes and the development of baseline reference sites:
  - Increase the number of depth intervals samples within a given depth, e.g., 0–10 cm, 10–20 cm, and 20–30 cm for 30 cm depth.
  - Use the published tool of Von Haden et al.^34^.
  - Consider deeper sampling cores^21,36^.

Based on our results and rationale, and incorporating the necessary tradeoffs between cost and accuracy, recommendation 3 is a realistic option. The use of our ESM corrective approach with a single split sample depth will segregate the gains in SOC stock and changes in BD to adequately correct for the error associated with changes in BD.

## Conclusions

Correcting for the use of fixed depth sampling for more accurate estimation of SOC stock and SOC stock changes is needed. The ESM corrective approach with split depth sampling and a simple linear interpolation correction allows for a tradeoff of accuracy and cost. The results generated from this approach have the potential to help incentivize environmentally beneficial management practice changes, reward farmers, provide more accurate estimates of SOC stock and its changes, and help validate the legitimacy of the accounting practices used by the emerging carbon market and organizations that have pledged to reduce their supply chain GHG footprints to improve soil, water, and air quality.

## Supplementary Information

Supplementary Information 1.

Supplementary Information 2.

## Data Availability

All simulation assumptions and calculations are contained in this text, references here in, and in supplementary information.

## Abbreviations

GHG: Greenhouse gas emissions
SOC: Soil organic carbon
BD: Bulk density
D_a_: Adjusted soil depth
