# Supplementary material for: A simple soil mass correction for a more accurate determination of soil carbon stock changes
Source: Sci Rep. 2023 Feb 8;13:2242. doi: 10.1038/s41598-023-29289-2 (PMC9908890; doi:10.1038/s41598-023-29289-2)
Supplement: Supplementary file 1 — Supplementary Information 1. [file 41598_2023_29289_MOESM1_ESM.docx]

# Supplemental information 1:

The R script to build, sample and correct bulk density (BD) and subsequent soil organic carbon (SOC) stocks from a theoretical 30cm sample is presented in three flies: main.R, functions.R, and plots.R.

## Main.R

#### main program -----

## source functions

source("./R/corrections/functions.R")

### 1) Build theoretical Data set ------------------------

#InitialIZE soils for each scenario

dt_s1_t0 <- calc_soil (soc= soc, soc_30= 1.4, soc_linear=TRUE, BD_30=1.5, BD_linear=TRUE ) %>% mutate(scenario = "s1", period= "t0")

dt_s2_t0 <- calc_soil (soc= soc, soc_30= 1.4, soc_linear=FALSE, BD_30=1.5, BD_linear=FALSE ) %>% mutate(scenario = "s2", period= "t0")

# Expand base soil t1

dt_s1_t1 <- expand_soil(dt_s1_t0, BD_new = 1.3, soc_new = 1.5) %>% mutate(scenario = "s1", period= "t1")

dt_s2_t1 <- expand_soil(dt_s2_t0, BD_new = 1.3, soc_new = 1.5) %>% mutate(scenario = "s2", period= "t1")

# Expand base soil t2

dt_s1_t2 <- expand_soil(dt_s1_t1, BD_new = 1.1, soc_new = 1.6) %>% mutate(scenario = "s1", period= "t2")

dt_s2_t2 <- expand_soil(dt_s2_t1, BD_new = 1.1, soc_new = 1.6) %>% mutate(scenario = "s2", period= "t2")

# Combine all expanded base soil and name.

t_list <- list(dt_s1_t0, dt_s2_t0, dt_s1_t1, dt_s2_t1, dt_s1_t2, dt_s2_t2)

names(t_list) <- c("dt_s1_t0", "dt_s2_t0", "dt_s1_t1", "dt_s2_t1", "dt_s1_t2", "dt_s2_t2")

t_dt <- rbindlist(t_list,use.names=TRUE)

# Sample all soils and combine

sample_10cm <- map(t_list,function(H) sample_soil(interval = 10, H))

sample_15cm <- map(t_list,function(H) sample_soil(interval = 15, H))

sample_30cm <- map(t_list,function(H) sample_soil(interval = 30, H))

sample_list <- c(sample_10cm, sample_15cm, sample_30cm)

sample_dt <- rbindlist(sample_list)

### 2. Correct all samples ------------------------------------------------

# see function correct_soil in functions.R for equations in the manuscript.

# manually list all scenarios and hypothetical curves for plotting

sampled_scenarios <- list(

sample_30cm$dt_s1_t0[,7:14] %>% mutate(correction = "Sampled") ,

sample_30cm$dt_s2_t0[,7:14] %>% mutate(correction = "Sampled") ,

correct_soil(intial_soil = sample_30cm$dt_s1_t0, sample = sample_30cm$dt_s1_t1),

correct_soil(intial_soil = sample_30cm$dt_s1_t0, sample = sample_30cm$dt_s1_t2),

correct_soil(intial_soil = sample_30cm$dt_s2_t0, sample = sample_30cm$dt_s2_t1),

correct_soil(intial_soil = sample_30cm$dt_s2_t0, sample = sample_30cm$dt_s2_t2),

sample_15cm$dt_s1_t0[,7:14] %>% mutate(correction = "Sampled") ,

sample_15cm$dt_s2_t0[,7:14] %>% mutate(correction = "Sampled") ,

correct_soil(intial_soil = sample_15cm$dt_s1_t0, sample = sample_15cm$dt_s1_t1),

correct_soil(intial_soil = sample_15cm$dt_s1_t0, sample = sample_15cm$dt_s1_t2),

correct_soil(intial_soil = sample_15cm$dt_s2_t0, sample = sample_15cm$dt_s2_t1),

correct_soil(intial_soil = sample_15cm$dt_s2_t0, sample = sample_15cm$dt_s2_t2),

sample_10cm$dt_s1_t0[,7:14] %>% mutate(correction = "Sampled") ,

sample_10cm$dt_s2_t0[,7:14] %>% mutate(correction = "Sampled") ,

correct_soil(intial_soil = sample_10cm$dt_s1_t0, sample = sample_10cm$dt_s1_t1),

correct_soil(intial_soil = sample_10cm$dt_s1_t0, sample = sample_10cm$dt_s1_t2),

correct_soil(intial_soil = sample_10cm$dt_s2_t0, sample = sample_10cm$dt_s2_t1),

correct_soil(intial_soil = sample_10cm$dt_s2_t0, sample = sample_10cm$dt_s2_t2))

sampled_scenarios_dt <- sampled_scenarios %>% rbindlist(.,use.names=TRUE )

## build out error

total_SOC_stock_sampled <- sampled_scenarios_dt %>%

group_by(scenario, period,interval) %>%

slice(which.max(depth))

# get values at the same mineral mass as t0 for all scenario and periods

getit <- map(t_list, function(H){

approx(x = H$Min_mass_soil_MG_ha, y = H$SOC_Stock_MG_ha, xout =(total_SOC_stock_sampled$Min_mass_soil_MG_ha %>% first), method = "linear",

na.rm = TRUE)$y})

# get scenario and period lables

indexs <- map(t_list, function(H){

temp_out <- H %>% dplyr::select(scenario, period) %>% unique

}) %>% do.call("rbind", .)

# Build data frame

names(getit) <- NULL

ddd <- as.data.frame(getit) %>% t

row.names(ddd)=NULL

ddd = as.data.frame(x=list("SOC_Stock_MG_ha_global" = ddd))

index2 <- cbind(indexs, ddd)

total_SOC_stock_sampled <- merge(total_SOC_stock_sampled, index2)

### 3. Calculate Error ------------------------------------------------

## Error due to sample size

total_SOC_stock_sampled <- total_SOC_stock_sampled %>%

mutate(error = (SOC_Stock_MG_ha-SOC_Stock_MG_ha_global)/SOC_Stock_MG_ha_global)

## Error from no correction the mineral mass SOC@30cm

total_SOC_stock_sampledonly<- sampled_scenarios_dt %>%

group_by(scenario, period,interval) %>% filter(correction=="Sampled") %>%

slice(which.max(depth))

A <- (total_SOC_stock_sampledonly %>% filter(interval ==30, period =="t0"))$SOC_Stock_MG_ha

B <- (total_SOC_stock_sampledonly %>% filter(interval ==30, period =="t2"))$SOC_Stock_MG_ha

no_correction <- as.data.frame(list(interval = "No correction", error = ((B-A)/A), scenario = c("s1","s2")))

error_join <- rbind(total_SOC_stock_sampled %>%
 select(names(no_correction)),no_correction, fill = TRUE, use.names = TRUE)

## functions.R

#####################################

# Functions for BD corrections #

# Author: Ames Fowler #

# Date: 8/10/2022 #

#####################################

# get libraries

library(tidyverse)

library(data.table)

# Guess initial values

v = c(1,.8,-.04)

# SOC stock percentages from Jobbagy & Jackson 2000

target_values <- c(41, 23, 15, 12, 9)

# depth vector for Jobbagy & Jackson 2000

vectora <- seq(20, 100, 20)

#function to calculate SOC curve

fn <- function(v) {

soc_o = v[1]

soc_inf = v[2]

k = v[3]

vectorc <- soc_inf+(soc_o-soc_inf)*exp(k*vectora)

vectorc <- vectorc/sum(vectorc)*100

return <- sum(abs(target_values - vectorc))

}

#R solver for SOC curve parameters

solve_out <- optim(v, fn)

# get SOC curve perameters

soc_o = solve_out$par[1]

soc_inf = solve_out$par[2]

k = solve_out$par[3]

#Build the hypotheical soil profile

depth = seq(0,80,1)

soc <- soc_inf+(soc_o-soc_inf)*exp(k*depth)

soc_0_ratio <- soc[1]/soc[2]

# Funct. Build hypothetical soil-----

# based on provided average soc (soc_30) and BD (BD_30) values and a soc profile

# if soc_linear is false (SOC decay inacted)

calc_soil <- function(soc= soc, soc_30, soc_linear=FALSE, BD_30, BD_linear ){

if(soc_linear == FALSE){

# scale the soc profile curve to soc_30 average

soc_scale <- soc_30/mean(soc[2:31])

soc <- soc * soc_scale

}else{

soc = soc_30

}

if(BD_linear == FALSE){

# build the BD sieres with =+/- 10%

BD = BD_30+seq(-1,1,2/29)*BD_30*.1

W = BD/30

soc[2:31] = mean(W)/W*soc[2:31]

# with changing BD there is transient mass.

# To ensure the same 30m mass percentage the SOC

# percentage needs to be weighted up with less BD

soc[1] = soc[2]*ifelse(soc_linear==FALSE,soc_0_ratio,1)

BD = c(BD[1], BD, rep(BD[30], 50))

}else{

BD = BD_30#1.5

}

dt<- as.data.frame(cbind(depth, BD, soc, soc_linear, BD_linear))

# Calculate all the soil values from depth, BD, and SOC

temp_dt <- dt %>% mutate(depth_inc = depth - lag(depth) %>% replace_na(0),

soc_30 = sum(BD[2:31]*soc[2:31])/sum(BD[2:31]),

T_soil_mass_Mg_ha_inc = depth_inc*BD*10^3/10,

Min_mass_soil_MG_ha_inc = T_soil_mass_Mg_ha_inc*(1-1.9*soc/100),

T_soil_mass_Mg_ha = T_soil_mass_Mg_ha_inc %>% cumsum,

Min_mass_soil_MG_ha = Min_mass_soil_MG_ha_inc %>% cumsum,

SOC_Stock_MG_ha_inc = T_soil_mass_Mg_ha_inc*soc/100,

SOC_Stock_MG_ha = SOC_Stock_MG_ha_inc%>% cumsum)

}

##for testing

# dt = dt_s2_t0

# soc_new = 1.5

# BD_new = 1.3

# Funct. expand soil ----

expand_soil <- function(dt, BD_new, soc_new){

#explained soil depth by type

# BD_new = 1.1

BD_mean = mean(dt$BD[2:31])

#calculate the depth of the old soil that fills 30cm with new BD average.

DA = 30 *BD_new/BD_mean

if( dt$BD_linear %>% first == 0){

# calc percent change.

prct_change = BD_mean/BD_new - 1

# build the new BD sequence

BD_seq = BD_new*(1-seq(prct_change, -prct_change, -prct_change*2/(DA)))

# add in the zero point and values greater than 30

BD_seq_out <- c(BD_seq[1]-(BD_seq[2] -BD_seq[1]), BD_seq,rep(BD_seq[length(BD_seq)], ceiling(80-DA-1)))

}else{

# If not varying with depth... all is constant.

BD_seq= rep(BD_new, 81)

BD_seq_out= rep(BD_new, 81)

}

# calculate the depth series of teh expanded 1cm increments, now some value 1+cm for each.

Depth_out = c(0,dt$BD[2:length(dt$BD)]/BD_seq_out[2:length(dt$BD)]) %>% cumsum

# interpolate BD values for new profile (back to 1:30 depth by 1cm)

temp <- approx(x = Depth_out, y = BD_seq_out, xout = dt$depth, method = "linear",

yright =BD_seq[length(BD_seq)], ties = mean, na.rm = TRUE) %>%

as.data.frame()

names(temp) <- c("depth", "BD")

# mean(temp$BD[2:31]) ## for checking

# interpolate SOC values for new profile (back to 1:30 depth by 1cm)

temp2 <- approx(x = Depth_out[1:81], y = dt$soc, xout = dt$depth, method = "linear",

yright =BD_seq[length(BD_seq)], ties = mean, na.rm = TRUE) %>%

as.data.frame()

names(temp2) <- c("depth", "soc")

### merge all

temp = merge(temp, temp2) %>% cbind( dt[,c("soc_linear", "BD_linear", "scenario")])

#sum(temp$soc[2:31]*temp$BD[2:31])/sum(temp$BD[2:31]) ###check average SOC

#correct SOC to fixed level

if(first(temp$soc_linear) ==0){

## find the net change in SOC stock from the expanded soil from the 30cm Base line

net_soc_stock = soc_new*mean(temp$BD[2:31])*30 - sum(temp$soc[2:31]*temp$BD[2:31])

## weight by SOC stock by SOC distribution and

## multiple by BD to convert back to SOC concentration

net_soc = net_soc_stock*soc[2:31]/mean(soc[2:31])/30/temp$BD[2:31]

## add the increase in SOC and fix the zero value

temp$soc[2:31] = c((net_soc))+temp$soc[2:31] #,rep(0,20)

temp$soc[1] = temp$soc[2]*ifelse(first(temp$soc_linear)==0,soc_0_ratio,1)

# sum(temp$soc[2:31]*temp$BD[2:31])/sum(temp$BD[2:31]) ## check average

}else{

temp$soc = soc_new

}

#recalucate the soil variables.

temp_dt <- temp %>% mutate(depth_inc = depth - lag(depth) %>% replace_na(0),

soc_30 = sum(BD[2:31]*soc[2:31])/sum(BD[2:31]),

T_soil_mass_Mg_ha_inc = depth_inc*BD*10^3/10,

Min_mass_soil_MG_ha_inc = T_soil_mass_Mg_ha_inc*(1-1.9*soc/100),

T_soil_mass_Mg_ha = T_soil_mass_Mg_ha_inc %>% cumsum,

Min_mass_soil_MG_ha = Min_mass_soil_MG_ha_inc %>% cumsum,

SOC_Stock_MG_ha_inc = T_soil_mass_Mg_ha_inc*soc/100,

SOC_Stock_MG_ha = SOC_Stock_MG_ha_inc%>% cumsum)

}

# Funct. Samples soils ---------

# given a soil and sample depth interval

# this function produces aggregated depth samples.

sample_soil <- function(interval, soil){

# Split soils to relavent parts.

soil0 <- soil[1,]%>%

mutate( ints = 0, scenario = first(scenario),

period= first(period),

interval = interval)

soil1 <- soil[2:31,]

soil3 <- soil[32:81,]

#build intervals if sample depth is less than 30

if((30/interval)>1){

soil1 <- soil1 %>%

mutate( ints = cut(depth, breaks =(30/interval)) %>% as.integer())

}else{

soil1 <- soil1 %>%

mutate( ints = 1)

}

## group by intervals (ints) and sum mass values

##

soil_sum <- soil1 %>%

group_by(ints) %>%

summarise(depth_inc = sum(depth_inc),

Min_mass_soil_MG_ha_inc = sum(T_soil_mass_Mg_ha_inc*(1-1.9*soc/100)),

T_soil_mass_Mg_ha_inc = sum(T_soil_mass_Mg_ha_inc),

SOC_Stock_MG_ha_inc = sum(SOC_Stock_MG_ha_inc),

BD = T_soil_mass_Mg_ha_inc/depth_inc/10^3,

soc = SOC_Stock_MG_ha_inc/T_soil_mass_Mg_ha_inc*100,

scenario = first(scenario),

period= first(period),

interval = interval) %>% ungroup %>%

mutate(depth = cumsum(depth_inc),

Min_mass_soil_MG_ha = cumsum(Min_mass_soil_MG_ha_inc),

SOC_Stock_MG_ha = cumsum(SOC_Stock_MG_ha_inc),

T_soil_mass_Mg_ha = cumsum(T_soil_mass_Mg_ha_inc),

soc_30 = sum(BD*soc)/sum(BD))

## add back in the zero sample for plotting.

sum_soil_out <- rbind(soil0 %>% dplyr::select(names(soil_sum)), soil_sum)

}

# intial_soil = sample_10cm[[3]]

# sample = sample_10cm[[6]]

# ## correction function

#Funct. Correct soil ----

#

correct_soil <- function(intial_soil, sample){

Dj <- sample$interval %>% first() #interval depth

# Drop the Zero level

intial_soil <- intial_soil[-1,]

sample2 <- sample[-1,]

ns <- nrow(sample2) #number of intervals.

# Calculate the change in soil depth from the last layer eq.7

DA = sample2$depth[ns]+

(Dj*sum(intial_soil$BD* (1-1.9*intial_soil$soc/100))-Dj*sum(sample2$BD* (1-1.9*sample2$soc/100)))/

(sample2$BD[ns]* (1-1.9*sample2$soc[ns]/100))

# find the change is soil depth. Eq.4

delta_D <- DA-sample2$depth[ns]

# Calculate the corrected total SOC stock Eq.5

SOC_Stock_MG_ha <- (Dj*sum(sample2$BD*sample2$soc)+

delta_D*sample2$BD[ns]*sample2$soc[ns])*10

# Calculate the corrected total soil mass

T_soil_mass_Mg_ha <- (Dj*sum(sample2$BD)+

delta_D*sample2$BD[ns])*10^3

# Calculate the corrected total mineral mass

Min_mass_soil_MG_ha <- (Dj*sum(sample2$BD* (1-1.9*sample2$soc/100))+

delta_D*sample2$BD[ns]* (1-1.9*sample2$soc[ns]/100))*10^3

# Collect the corrected soil values

temp = as.data.frame(list("T_soil_mass_Mg_ha" = T_soil_mass_Mg_ha, "Min_mass_soil_MG_ha" = Min_mass_soil_MG_ha,"SOC_Stock_MG_ha"= SOC_Stock_MG_ha, depth = DA))

# Add the corrected soils values to the sampled soil, duplicate the 30cm layer for plotting.

# lable the

temp_out <- rbind(sample[,c(11:14)],sample[ns+1,c(11:14)], temp) %>%

mutate(soc = c(sample$soc,NA, NA),

scenario = unique(sample$scenario),

period = unique(sample$period),

interval = unique(sample$interval),

correction = c(rep("Sampled", ns+1), "Corrected","Corrected"))

}

## Plots.R

# plotting

fig_path <- "C:/Users/fowler53/Michigan State University/EESBassoLab - Ames small projects/Bulk Density/BD correction/figures"

# Scenario plot: figure 2

g1 <- t_dt %>% filter(scenario != "temp") %>% ggplot()+

geom_line(aes(y = depth, x = BD, col = period), size =1)+

facet_grid(col = vars(scenario))+

theme_minimal()+scale_y_reverse(limits =c(30,0))+

scale_colour_brewer(palette = 7)+

geom_segment(aes(x = 1.175, y = 10, xend = 1.125, yend = 10),

arrow = arrow(length = unit(0.15, "cm")), size =1)+

geom_segment(aes(x = 1.4, y = 10, xend = 1.35, yend = 10),

arrow = arrow(length = unit(0.15, "cm")), size =1) +

labs(x = "Bulk Density",y="Depth", col = "Time \nperiod")

ggsave(g1 , filename = file.path(fig_path, "BD_scenarios_2.jpeg"),

width = 5, height = 3, dpi = 700, units="in")

g2 <- t_dt %>% filter(scenario != "temp") %>%

ggplot()+geom_line(aes(y = depth, x = soc, col = period), size =1)+

facet_grid(col = vars(scenario))+

theme_minimal()+scale_y_reverse(limits =c(30,0))+

scale_colour_brewer(palette = 5)+

labs(x = "SOC",y="Depth", col = "Time \nperiod")

ggsave(g2 , filename = file.path(fig_path, "SOC_scenarios_2.jpeg"),

width = 5, height = 3, dpi = 700, units="in")

sampled_scenarios_dt$interval <- paste0(sampled_scenarios_dt$interval,"cm")

t_dt$interval = "S2 Baseline"

t_dt$correction = "Sampled"

test_bind <- rbind(sampled_scenarios_dt, t_dt, fill = TRUE)

color_pallet <- c("#F8766D", "#7CAE00", "#00BFC4", "grey50")

inital_min_mass <- (t_dt %>%

filter(scenario=="s1", period =="t0"))$Min_mass_soil_MG_ha [31]

g4 <- test_bind %>% filter(scenario=="s2") %>%

ggplot(.)+geom_line(aes(x = Min_mass_soil_MG_ha , y = SOC_Stock_MG_ha,

col = interval %>%

factor(levels = c("30cm","15cm","10cm", "S2 Baseline")),

linetype = correction %>%

factor(levels = c("Sampled", "Corrected"))),

alpha = .5)+

facet_grid(cols = vars(period), rows = vars(scenario))+

theme_minimal()+theme(legend.position="bottom")+

labs(x = "\nCummulative mineral mass (Mg/ha)",

y ="Cummulative SOC stock (Mg/ha)\n",

col = "Sample depth interval", linetype = "Data type")+

scale_color_manual(values = color_pallet)

l <- coord_cartesian(ylim=c(0,75),xlim=c(0, inital_min_mass))

g4.1 <- geom_point(data = test_bind %>%

filter(interval!="S2 Baseline", scenario=="s2"),

aes(x = Min_mass_soil_MG_ha , y = SOC_Stock_MG_ha,

col = interval %>%

factor(levels = c("30cm","15cm","10cm", "S2 Baseline"))),

alpha=.5)

ggsave(g4+g4.1+l, filename = file.path(fig_path, "SOC_mass_curve.jpeg"),

width = 8.5, height = 4, dpi = 700, units="in")

# s2 t2 cut out: figure 6

g4.5 <- test_bind %>% filter(scenario=="s2", period == "t2") %>%

ggplot(.)+geom_line(aes(x = Min_mass_soil_MG_ha , y = SOC_Stock_MG_ha,

col = interval %>%

factor(levels = c("30cm","15cm","10cm", "S2 Baseline")),

linetype = correction %>% factor),

alpha = .5)+

facet_grid(rows = vars(scenario), col = vars(period))+

theme_minimal()+theme(legend.position="bottom")+

labs(x = "\nCummulative mineral mass (Mg/ha)",

y ="Cummulative SOC stock (Mg/ha)\n",

col = "Sample depth interval", linetype = "Data type")+

scale_color_manual(values = color_pallet)+

scale_linetype(guide = "none")

l2 <- coord_cartesian(ylim=c(50,75),xlim=c(3000, inital_min_mass))

l_no_correction <- geom_segment(aes(x = inital_min_mass, y = 52,

xend = inital_min_mass, yend = 63.1),

col = color_pallet[4])

l_30 <- geom_segment(aes(x = inital_min_mass+30, y = 72.3,

xend = inital_min_mass+30, yend = 63.1),

col = color_pallet[1])

l_15 <- geom_segment(aes(x = inital_min_mass+15, y = 68,

xend = inital_min_mass+15, yend = 63.1),

col = color_pallet[2])

l_10 <- geom_segment(aes(x = inital_min_mass, y = 66.7,

xend = inital_min_mass, yend = 63.1),

col = color_pallet[3])

g4.5+l2+l_no_correction+l_30+l_15+l_10

ggsave(g4.5+l2+l_no_correction+l_30+l_15+l_10,

filename = file.path(fig_path, "ex_error.jpeg"),

width = 6, height = 4.25, dpi = 700, units="in")

## error figure

t2 <- no_correction%>% filter(scenario == "s2") %>% mutate(period = "t2")

t1 <- total_SOC_stock_sampled %>% filter(scenario == "s2", period == "t2") %>%

select(names(t2))

t1$interval <- paste0(t1$interval,"cm")

t3 <- rbind(t1,t2)

t3$interval[t3$interval == "No correction"] ="Fixed depth"

gg <- t3 %>%

ggplot()+

geom_col(aes(x = interval %>%

factor(levels = c("30cm","15cm","10cm", "Fixed depth")),

y = error,

fill = interval %>%

factor(levels = c("30cm","15cm","10cm", "Fixed depth"))))+

theme_minimal()+

facet_grid(cols = vars(scenario))+

labs(y = "Percent error\n", x = "Sample depth interval",

fill = "Sample depth interval")+

theme(legend.position="bottom")+

theme(axis.title.x=element_blank(),

axis.text.x=element_blank(),

axis.ticks.x=element_blank())+

geom_text(

aes(label = error %>% round(2), x = interval %>%

factor(levels = c("30cm","15cm","10cm", "Fixed depth")),

y = error *1.1),

position = position_dodge(0.9),

vjust = 0

)+scale_fill_manual(values = color_pallet)

ggsave(gg, filename = file.path(fig_path, "s2_s3_error.jpeg"),

width =6, height = 4.5, dpi = 700, units="in")

# Supplemental information 2:

The excel document “Correction Example.xls” attached provides two sheets. The sheet “S1 simple correction example” provides an overview of the corrections for a single fixed depth sample. The sheet “S2 15cm depth interval example” demonstrates the application of the correction on a sample (0-30cm) with two depth intervals of 15 cm (0-15, and 15-30cm).
